# Effects of Mindfulness Therapy on Stress Biomarkers and Psychological Symptoms in Women With Recurrent Pregnancy Loss: A Randomized Clinical Trial

**DOI:** 10.1002/smi.70228

**Published:** 2026-10-14

**Authors:** Elaine C. F. Oliveira, Sara P. C. Paiva, Enrrico Bloise, Fernando M. Reis

**Affiliations:** ^1^ Department of Obstetrics and Gynecology Universidade Federal de Minas Gerais Belo Horizonte Brazil; ^2^ Department of Oncology Georgetown University Medical Center Washington DC USA; ^3^ Department of Morphology Universidade Federal de Minas Gerais Belo Horizonte Brazil

**Keywords:** cortisol, depression, mindfulness, recurrent miscarriage, recurrent pregnancy loss, salivary biomarkers, stress

## Abstract

Women with recurrent pregnancy loss (RPL) exhibit high levels of depression, anxiety, and stress, which negatively impact their quality of life. This randomized clinical trial evaluated the effects of an eight‐week online Mindfulness‐Based Intervention (MBI), with weekly one‐hour sessions, on psychological and biological markers of stress in 35 women with RPL. Participants were randomly assigned to either the MBI or standard care (control group) and assessed before and after the intervention through salivary biomarkers (cortisol, alpha‐amylase, and Immunoglobulin A—IgA) and the validated questionnaires Depression, Anxiety and Stress Scale‐21 (DASS‐21) and Psychological General Well‐Being Index (PGWB‐I). No significant time × group interactions were observed for salivary biomarkers, including cortisol (*p* = 0.672), alpha‐amylase (*p* = 0.342), and IgA (*p* = 0.148). Participants in the MBI group showed significant reductions in total DASS‐21 (*p* = 0.018), depression (*p* = 0.026), and stress (*p* = 0.015) scores, whereas anxiety did not differ significantly between groups (*p* = 0.121). They also showed robust improvements in overall well‐being (*p* < 0.001) and multiple PGWB‐I domains. These findings suggest that MBI may improve self‐reported psychological well‐being in women with RPL; however, as no significant effects were observed in the primary biomarker outcomes, these secondary psychological findings should be considered exploratory and hypothesis‐generating.

**Trial Registration:**

https://ensaiosclinicos.gov.br/rg/RBR‐4xc76cr

## 1 Introduction

Recurrent pregnancy loss (RPL) is traditionally defined as the occurrence of three or more consecutive pregnancy losses before 20 weeks of gestation (Rai and Regan 2006), although more recent guidelines consider two or more losses sufficient for diagnosis (Tulandi and Al‐Fozan 2024). It affects approximately 1% of couples trying to conceive when defined by a minimum of three losses, and about 3% when the definition includes two or more losses (Rai and Regan 2006).

Miscarriage is associated with an increased risk of mental health disorders, including depression and anxiety. It is estimated that up to two‐thirds of women with RPL experience some form of psychiatric illness (Klock et al. 1997). Psychological distress may persist for extended periods and often intensifies with successive pregnancy losses (Bergner et al. 2008; Carp 2014). Studies have confirmed that women with RPL tend to exhibit high levels of depression, anxiety, and stress, with a marked negative impact on their quality of life (He et al. 2019; Hedegaard et al. 2021; Kolte et al. 2015).

In this context, mindfulness‐based interventions (MBIs) have emerged as promising strategies to promote emotional well‐being and improve quality of life in women affected by RPL (Jensen et al. 2021; D. N. Wang et al. 2021). Mindfulness is defined as a meditative practice that involves maintaining a moment‐by‐moment awareness of thoughts, emotions, and bodily sensations with a non‐judgmental attitude (Kabat‐Zinn 2003). It typically includes exercises that help individuals become more aware of their internal experiences, promoting emotional regulation through slow, conscious breathing (Gasnier et al. 2017). Over the past decades, MBIs have been extensively explored as tools for enhancing well‐being in various populations, including women with RPL (Jensen et al. 2021; D. N. Wang et al. 2021).

The assessment of salivary biomarkers, such as cortisol, alpha‐amylase, and immunoglobulin A (IgA), offers non‐invasive, reliable methods to monitor physiological changes associated with stress‐related psychological conditions (Blair et al. 2017; Chojnowska et al. 2021; Gröschl 2008). The relationship between mindfulness and stress‐related biomarkers has been the focus of growing research (Lynch et al. 2011). These biomarkers reflect activity in both the immune system and the autonomic nervous system, and are regulated by the hypothalamic‐pituitary‐adrenal (HPA) axis, which plays a central role in the biological stress response (Chojnowoska et al. 2021; Engeland et al. 2019).

Cortisol is a glucocorticoid hormone secreted by the adrenal cortex in response to activation of the HPA axis. Under stress, the hypothalamus releases corticotropin‐releasing hormone (CRH), which stimulates the pituitary gland to secrete adrenocorticotropic hormone (ACTH), leading to cortisol production (Blair et al. 2017; Kirschbaum and Hellhammer 1989). Free cortisol diffuses passively through the membranes of the salivary glands and is secreted into the saliva. Thus, salivary cortisol concentration directly reflects its free and biologically active fraction, providing a reliable measure of the hormone levels available in the body (Blair et al. 2017; Gröschl 2008; Inder et al. 2012). Elevated salivary cortisol levels are often observed in individuals with chronic and acute stress and are commonly linked to depressive symptoms and anxiety (Blair et al. 2017; Gröschl 2008; Saraiva et al. 2005). According to meta‐analyses, MBIs have induced changes in salivary cortisol levels but the findings are inconsistent (Sanada et al. 2016; O'Leary et al. 2016; Vargas‐Uricoechea et al. 2024).

In turn, salivary alpha‐amylase is an enzyme regulated by the sympathetic nervous system. Its levels increase in response to acute stress due to heightened sympathetic activation. As a result, salivary alpha‐amylase has been recognized as a sensitive and rapid biomarker of autonomic nervous system arousal, complementing cortisol measurements in stress research, particularly in studies focused on anxiety and acute stress responses (Engeland et al. 2019; Chojnowska et al. 2021; Nater and Rohleder 2009). Research indicates that MBIs can effectively reduce salivary alpha‐amylase levels, suggesting reduced sympathetic nervous system activation (Aguilar‐Raab et al. 2021; Hassanvandi et al. 2025).

Secretory IgA is a key component of mucosal immune defense, especially in the oral and respiratory tracts (Chojnowska et al. 2021). Stress can modulate salivary IgA levels: acute stress typically increases its secretion, whereas chronic stress may have variable effects depending on the intensity and duration of exposure (Segerstrom and Miller 2004).

Therefore, the aim of this study was to evaluate salivary biomarkers of stress (cortisol, alpha‐amylase, and IgA), along with levels of depression, stress, anxiety, and general psychological well‐being in women with RPL before and after a MBI.

## 2 Methods

### 2.1 Study Approval and Registration

This randomized controlled trial (RCT) was approved by the Research Ethics Committee of Universidade Federal de Minas Gerais (Decision Number 5.895.786) and registered at the Brazilian Clinical Trials Registry, REBEC (Primary ID Number RBR‐4xc76cr) and Brazilian Ministry of Health Human Research Registry (Protocol ID 93675118.1.3001.5093).

Participation was entirely voluntary, and all participants provided written informed consent prior to enrollment.

### 2.2 Participants

Between July 2024 and March 2025, women aged 18 years or older with a history of at least two pregnancy losses before 20 weeks of gestation (excluding ectopic or molar pregnancies) under investigation for RPL at a tertiary academic hospital in Belo Horizonte, Brazil, were invited to participate in the research. These women were referred from various other hospitals and clinics in the region. Recruitment was conducted either in person, in a private setting, or via WhatsApp messaging.

All participants underwent a standard RPL workup according to the clinic's protocol, which included karyotyping of the couple, screening for antiphospholipid syndrome, serum Thyroid‐Stimulating Hormone (TSH), serum prolactin (if clinically indicated), transvaginal ultrasound, and hysteroscopy. Eligible participants were required to have internet access via mobile phone, computer, or tablet.

Exclusion criteria were diagnosis of chronic inflammatory or autoimmune disease, dementia, current smoking, inability to complete self‐administered questionnaires, being pregnant at the time of recruitment or prior experience with mindfulness practice or MBI programs.

### 2.3 Randomization, Allocation, and Concealment

Eligible participants who agreed to participate and signed the informed consent form were randomly assigned to either the intervention or the control group. Randomization was conducted using a computer‐generated sequence prepared in advance. Each participant then drew a numbered slip from a sealed opaque envelope, which corresponded to their group assignment. Thirty‐five participants were randomized (Figure 1).

**FIGURE 1:**
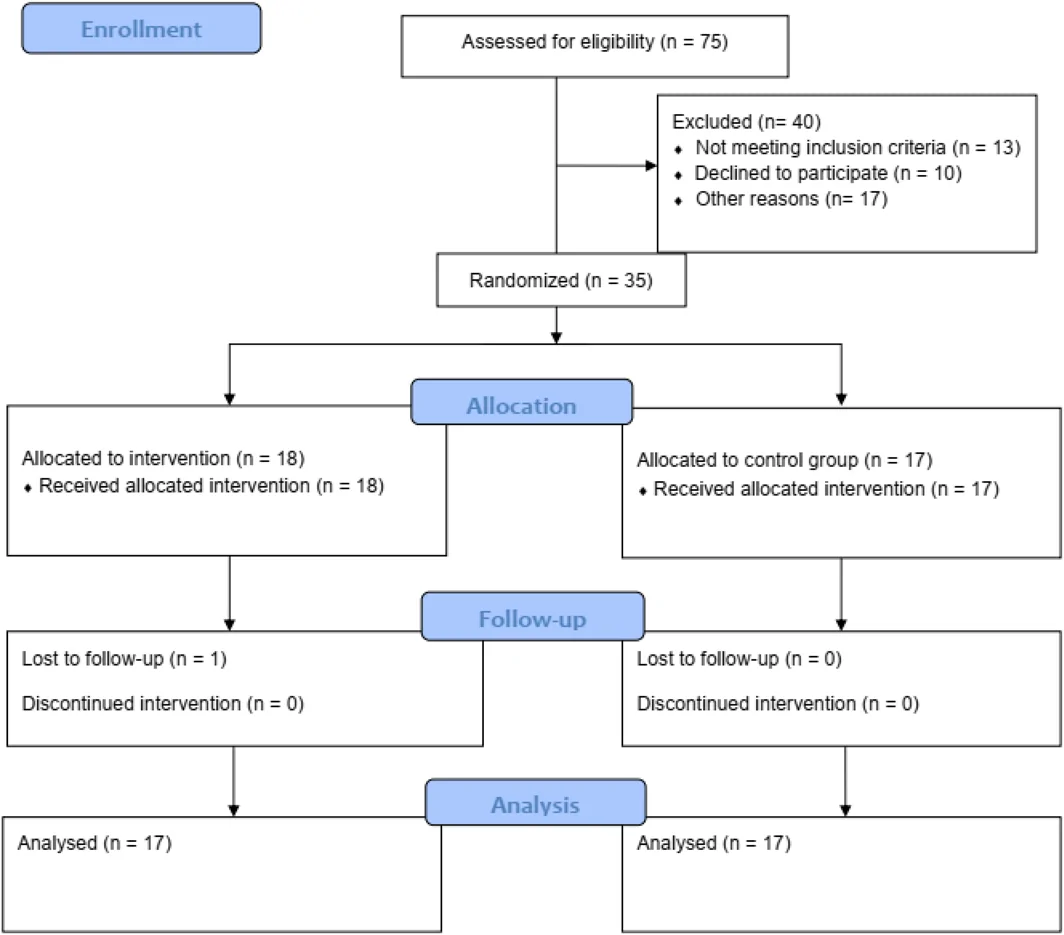
Flow diagram showing the selection, allocation, and follow‐up of the participants.

The intervention was open‐label (unblinded to participants and facilitators); however, outcome analysis was blinded. Self‐reported questionnaires and biochemical assays were assessed by evaluators blinded to group allocation.

### 2.4 Intervention

Women allocated to the intervention group participated in an 8‐week online MBI program delivered via Google Meet. The time interval between allocation and the first group meeting was 1 week. Sessions were held once weekly for 1 hour in synchronous format. The intervention followed a structured protocol based on the ‘Mind‐Body Therapy’ developed by Georgetown University (Saunders et al. 2007), which was previously validated in Brazil (Paiva et al. 2016). The sessions were conducted by an experienced therapist with over 15 years of mindfulness practice. Sessions included mindfulness meditation, relaxation, and visualization. Each group had up to five participants. The first session was introductory, with a brief meditation and explanation of the MBI approach. Subsequent sessions began with a short mindfulness meditation, followed by a ‘gratitude check‐in’ where participants shared something they were grateful for during the week. A mindfulness technique was then introduced and discussed. At the end of each session, participants received ‘homework,’ including mindful eating, healthy habits, journaling, and physical activity. A guided audio was also shared weekly to support individual practice.

Participants in the control group received standard care only, consisting of consultations with specialists and the routine diagnostic workup for RPL.

### 2.5 Study Outcomes

The primary outcome measures were changes in salivary concentrations of cortisol, alpha‐amylase, and IgA. Secondary outcomes were psychological symptoms of depression, stress and anxiety, and general well‐being, as measured by validated psychometric instruments. Saliva collection and questionnaire responses were performed 1 week before the start of the intervention and 1 week after its end.

### 2.6 Sample Collection and Processing

Saliva samples were collected in the morning, between 8:00 and 9:00 a.m., in person with the assistance of the researcher for all participants, using Salivette tubes (Sarstedt, Germany) and sterile containers to ensure standardized conditions and minimize contamination risk. Awakening time was not individually recorded. Salivary samples were collected at a single time point, which precluded dynamic cortisol assessments such as the cortisol awakening response, diurnal decline, or total daily output (Sanada et al. 2016).

For samples collected with Salivette devices, participants were instructed to keep the cotton swab in their mouth for approximately 3 minutes. For samples collected in sterile containers, participants were asked to slightly tilt their head forward, allowing saliva to accumulate naturally in the oral cavity, and to let it gently drip into the container without forceful spitting until a minimum volume of 2 mL was reached. Prior to collection, all participants were instructed to fast, refrain from drinking beverages (except water), and avoid brushing their teeth for at least 60 minutes, as well as to remain at rest for 1 hour before the procedure.

After collection, the samples obtained with Salivette devices were centrifuged for 2 minutes at 2500 × g and immediately frozen in the same tube at −20°C, where they remained stored until shipment to the laboratory for analysis. The samples collected in sterile containers were also kept frozen at −20°C until laboratory assays were performed. Saliva collected in Salivette tubes was used for salivary cortisol determination, whereas samples collected in sterile containers were used for the analysis of salivary IgA and alpha‐amylase levels.

#### 2.6.1 Salivary Cortisol Assay

Salivary cortisol concentrations were determined by electrochemiluminescence immunoassay (ECLIA) using the Elecsys Cortisol II kit on a Cobas e analyzer (Roche Diagnostics, Mannheim, Germany). Frozen saliva samples were analyzed individually with ready‐to‐use liquid reagents, requiring no manual preparation. System calibration was performed using a two‐point adjustment linked to a lot‐specific master curve automatically recognized by the reagent barcode. The assay's measurement range was 1.5–1750 nmol/L, with values below 1.5 nmol/L reported as undetectable. The analytical limits were: LoB = 1.0 nmol/L, LoD = 1.5 nmol/L, and LoQ = 3.0 nmol/L. Quality control procedures were conducted according to the manufacturer's recommendations, using PreciControl Universal and/or PreciControl Cortisol Saliva materials. Reported intra‐assay and inter‐assay coefficients of variation are 2%–6% and 3%–12%, respectively. For interpretation, results were compared with the reference salivary values provided in the manufacturer's insert: morning (6–10 a.m.), < 20.3 nmol/L (95th percentile); afternoon (4–8 p.m.), < 6 nmol/L; and midnight (± 30 min), < 7.56 nmol/L.

#### 2.6.2 Salivary Alpha‐Amylase Assay

Salivary alpha‐amylase concentrations were determined by spectrophotometry using a commercial assay kit on the AU5800 analyzer (Beckman Coulter). Samples were previously diluted at a ratio of 1:200, and final results were obtained after correction for the dilution factor. Calibration was performed according to the manufacturer's instructions, with the enzymatic activity of the calibrator being lot‐dependent (144 U/L for the lot used). The lower limit of detection was 10 U/L, and the assay sensitivity was 0.77 U/L. Analyses were conducted in single‐test mode, without replicates, yielding intra‐assay and inter‐assay coefficients of variation of 1.4% and 1.6%, respectively. For interpretation, results were compared with the reference range reported in the literature (80,000–625,000 U/L).

#### 2.6.3 Salivary Immunoglobulin A Assay

Salivary IgA concentrations were determined by nephelometry using a commercial assay kit on the Atellica analyzer (Siemens), as confirmed in the Siemens BN reference system. No prior extraction or dilution of samples was required. Analyses were performed in single‐test mode without replicates. Calibration was carried out according to the manufacturer's instructions, with standard curve values dependent on the calibrator lot. The minimum detectable concentration and assay sensitivity were both 6.0 mg/L (0.6 mg/dL). The intra‐assay coefficient of variation was 5.6%. For interpretation, results were compared with the reference range reported in the literature (1.59–41.45 mg/dL).

### 2.7 Psychometric Assessment

#### 2.7.1 Depression, Anxiety and Stress Scales

Depression, anxiety, and stress were assessed using the Depression, Anxiety and Stress Scale‐21 (DASS‐21), a 21‐item instrument that evaluates symptoms experienced over the previous seven days. The questionnaire comprises three subscales: depression, anxiety, and stress. Each subscale contains seven items rated on a 4‐point Likert scale from 0 (‘did not apply to me at all’) to 3 (‘applied to me very much or most of the time’). Scores for each subscale are summed and then multiplied by two, as recommended, to ensure comparability with the original 42‐item version (DASS‐42). Thus, each domain yields a total score ranging from 0 to 42, with higher scores indicating greater symptom severity (Lovibond and Lovibond 1995).

#### 2.7.2 General Well‐Being

Psychological well‐being was assessed using the Psychological General Well Being Index (PGWB‐I), which includes 22 items divided into six domains: anxiety, depressed mood, positive well‐being, self‐control, general health, and vitality. All items in this scale refer to symptoms and feelings experienced by the individual during the past month. In the Portuguese version of the questionnaire, each item is scored from 1 to 6, and total scores range from 22 to 132. Higher scores indicate greater psychological well‐being (Dupuy et al. 1984; Wenger et al. 1984; Dupuy et al. 1984; Espírito Santo 2015; Grossi et al. 2006).

### 2.8 Sample Size

The sample size calculation was performed during the study planning phase, based on the work by Lynch et al. (Lynch et al. 2011). The calculation aimed to detect a between‐group difference of 8.28 nmol/L in salivary cortisol levels (equivalent to 0.3 μg/dL in the original study), with a 95% confidence level and 80% statistical power. A minimum sample size of 16 participants per group was estimated, totaling 32 participants.

### 2.9 Statistical Analysis

Analyses were performed using complete‐case data, including participants with available pre‐ and post‐intervention assessments. The distribution of continuous variables was assessed using the Shapiro–Wilk normality test, from which the choice of statistical parametric or nonparametric methods was derived. Categorical variables were analyzed using the chi‐square test. Since some of the data did not follow a normal distribution, baseline group comparisons were performed using medians and the Mann–Whitney *U* test. For inferential analyses, variables with non‐normal distribution were log‐transformed when necessary to better satisfy model assumptions.

The effects of the MBI versus no intervention on the outcome variables were evaluated using repeated‐measures Analysis of Covariance (ANCOVA), considering the time × group interaction and adjusting for variables that differed between groups in the baseline. Because only two repeated measurements were included (pre‐ and post‐intervention), the sphericity assumption was inherently met, and no Mauchly's test or Greenhouse–Geisser correction was required. Effect sizes were calculated by the Cohen's *d* formula using means and standard deviations (Dunlop et al. 1996). The hypothesis of linear association between variables was tested using Spearman's rank correlation coefficient. We used Prism Graphpad version 6 and SPSS version 22 software packages. All tests were two‐tailed and *p* < 0.05 was considered statistically significant.

## 3 Results

### 3.1 Participant Flow

The CONSORT flowchart (Figure 1) illustrates the process of recruitment, allocation, follow‐up, and analysis of participants. Of the 75 women assessed for eligibility, 40 were excluded: 13 for not meeting the inclusion criteria (four were pregnant at the time of the invitation; one did not speak Portuguese; three had recently participated in mindfulness sessions; two had cognitive impairment; and two lacked access to Google Meet), 10 declined to participate, and 17 were excluded for other reasons (12 did not attend on the day of allocation and five did not respond to the invitation). The 35 eligible participants were randomized, with 18 allocated to the intervention group and 17 to the control group. All allocated participants received the corresponding intervention. In the intervention group, one participant was lost to follow‐up due to non‐return, while no discontinuations occurred in the control group. In total, 17 participants in each group were included in the final analysis.

### 3.2 Baseline Characteristics

Table 1 presents the clinical characteristics of the participants who initiated the study. No statistically significant differences were observed between the control and intervention groups regarding age, race, educational level, number of pregnancies, number of deliveries, number of miscarriages, presence of living children, baseline total scores of the PGWB‐I and DASS‐21 questionnaires, or baseline levels of salivary stress biomarkers.

**TABLE 1:** Clinical characteristics of the participants included in the study.^a^

	Control (*n* = 17)	MBI (*n* = 18)	*p* value
Age (years)	37.0 (35.0–41.5)	38.5 (33.2–40.0)	0.708^b^
Race			0.085^c^
White	2 (11.8%)	7 (38.9%)	
Black	2 (11.8%)	0 (0%)	
Mixed‐race	13 (76.4%)	11 (61.1%)	
Education level			0.303^d^
Incomplete elementary education	1 (5.9%)	0 (0%)	
Complete elementary education	0 (0%)	0 (0%)	
Complete high school education	13 (76.5%)	11 (61.1%)	
Incomplete higher education	0 (0%)	2 (11.1%)	
Complete higher education	3 (17.6%)	5 (27.8%)	
Number of pregnancies	3.0 (2.5–3.0)	4.0 (2.8–5.3)	0.072^b^
Number of deliveries	0 (0–1.0)	0 (0–1.0)	0.782^b^
Number of previous miscarriages	2.0 (2.0–3.0)	3.0 (2.0–4.0)	0.096^b^
Previous live child			0.443^c^
Yes	3 (17.6%)	6 (33.3%)	
No	14 (82.4%)	12 (66.7%)	
Baseline median DASS‐21	44.0 (19.0–69.0)	62.0 (38.5–103.0)	0.096^b^
Baseline median PGWB‐I	81.0 (69.0–93.5)	70.0 (58.7–83.0)	0.083^b^
Salivary cortisol (nmol/L)	6.2 (5.0–7.4)	6.5 (3.5–11.1)	0.628^b^
Salivary alpha‐amylase (U/L)	109 (79–232)	81 (51–188)	0.204^b^
Salivary IgA (mg/dL)	10.70 (6.75–14.90)	9.73 (5.88–16.63)	0.873^b^

Abbreviations: DASS‐21, Depression, Anxiety and Stress Scale; PGWB‐I, Psychological General Well‐Being Index.

^a^ Data are presented as medians (interquartile ranges) or *n* (%).

^b^ *p*‐values refer to the Mann–Whitney test.

^c^ *p*‐values refer to the chi‐square test.

^d^ *p*‐values refer to the Fisher's exact test.

### 3.3 Primary Outcomes: Salivary Cortisol, Alpha‐Amylase, and IgA

A significant main effect of time was observed for salivary cortisol (*p* = 0.021), indicating variation between assessment points (median change of 1.9 [*d* = −0.45] and 1.4 [*d* = −0.59] nmol/L in the MBI and control groups, respectively). However, the time × group interaction was not significant (*p* = 0.672), suggesting similar changes across groups (Figure 2A–B).

**FIGURE 2:**
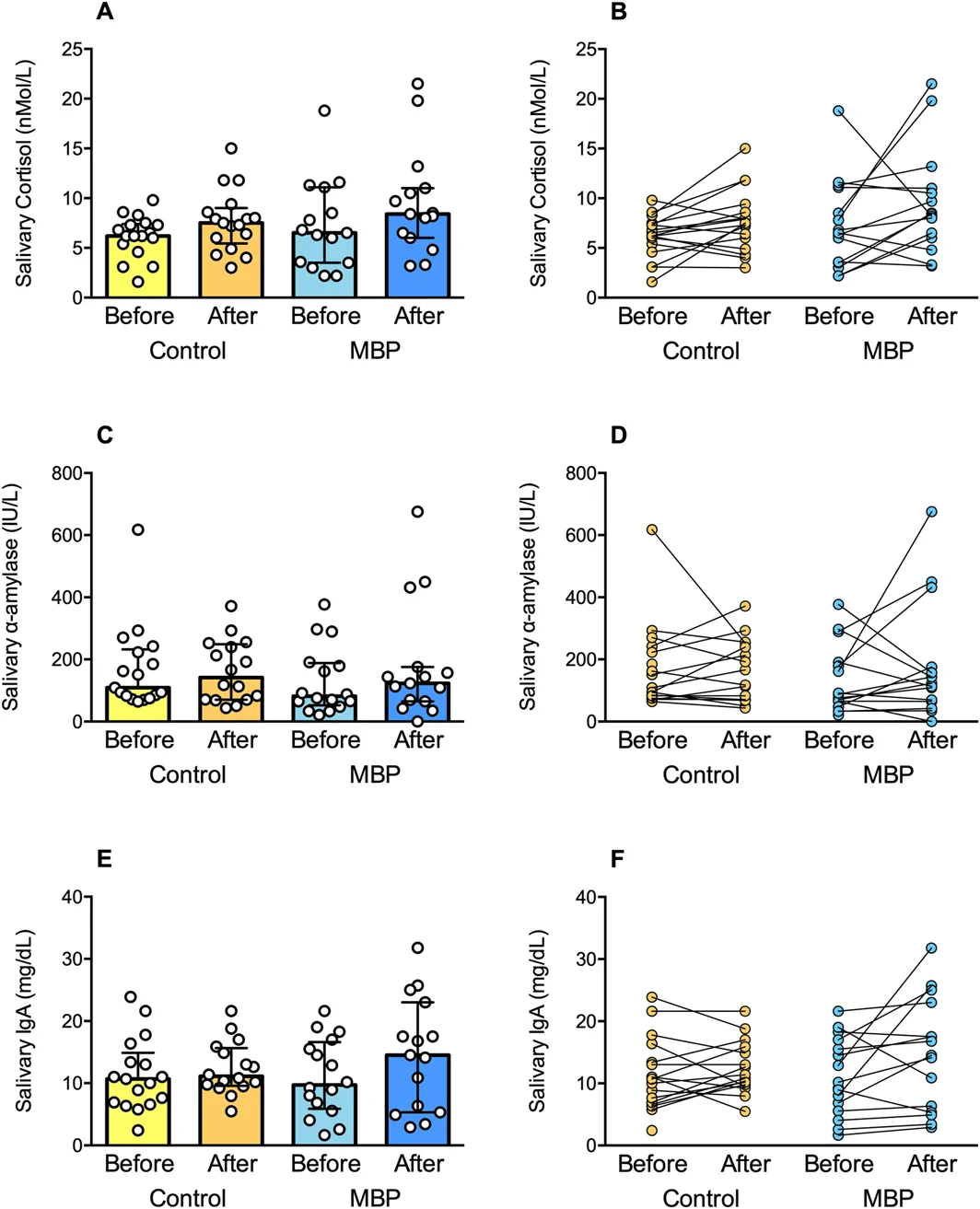
Changes in salivary biomarkers before and after the mindfulness‐based intervention (MBI) and in the control group. (A, B) Salivary cortisol (nmol/L) (MBI group: *n* = 15; control group: *n* = 17); (C, D) Salivary alpha‐amylase (U/L) (MBI group: *n* = 15; control group: *n* = 16); (E, F) Salivary immunoglobulin A (mg/dL) (MBI group: *n* = 15; control group: *n* = 16). Bars represent medians and interquartile ranges.

For salivary alpha‐amylase, neither the main effect of time (*p* = 0.478) nor the time × group interaction (*p* = 0.342) reached significance, indicating stable levels over time in both groups (Figure 2C–D).

Similarly, no significant main effect of time was found for salivary IgA (*p* = 0.061), and the time × group interaction was also nonsignificant (*p* = 0.148), showing comparable patterns between the intervention and control groups (Figure 2E–F).

### 3.4 Secondary Outcomes: Depression, Stress, and Anxiety, and General Well‐Being

Before the intervention, the intervention and control groups presented comparable levels of psychological distress (*p* = 0.096), with median total scores of 62 and 44, respectively (Figure 3A). The repeated‐measures ANOVA showed a significant main effect of time for total DASS‐21 scores (MBI group, *d* = 1.00; Control group, *d* = 0.21; *p* = 0.001), as well as for the depression (MBI group, *d* = 1.17; Control group, *d* = 0.27; *p* < 0.001), stress (MBI group, *d* = 1.18; Control group, *d* = 0.19; *p* = 0.001) and anxiety (MBI group, *d* = 0.56; Control group, *d* = 0.10; *p* = 0.036) subscales. A significant time × group interaction was observed for total scores (adjusted *p* = 0.014), indicating a greater reduction in symptoms in the MBI group (median total score = 24) compared with controls (median total score = 32, Figure 3A). When each domain was analyzed separately, this effect remained significant for depression (*p* = 0.026, adjusted *p* = 0.013) and stress (adjusted *p* = 0.048), but not for anxiety (adjusted *p* = 0.136) (Figure 3B–D).

**FIGURE 3:**
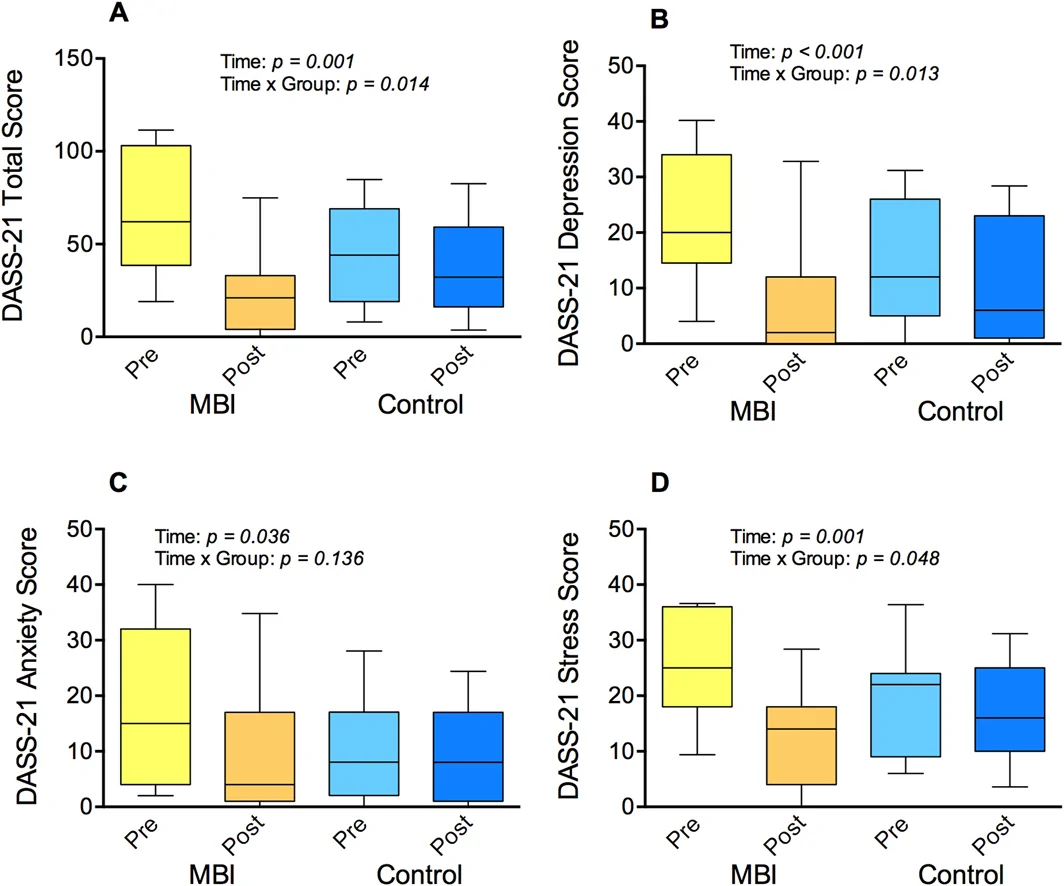
Changes in Depression, Anxiety, and Stress Scale (DASS‐21) scores before and after the mindfulness‐based intervention (MBI, *n* = 18 at baseline and *n* = 17 post‐intervention) and in the control group (*n* = 17 at baseline and post‐intervention). (A) Total score; (B) Depression; (C) Anxiety; (D) Stress. Boxes represent medians and interquartile ranges.

Regarding the PGWB‐I, baseline scores were 70 for the intervention group and 81 for the control group, increasing to 101 and 83, respectively, after the MBI (Figure 4). The repeated‐measures ANOVA revealed a significant main effect of time for total PGWB‐I scores (MBI group, *d* = −1.34; Control group, *d* = −0.20; *p* < 0.001) and for the domains of anxiety (*p* < 0.001), depression (*p* < 0.001), positive well‐being (*p* < 0.001), general health (*p* = 0.003), and vitality (*p* < 0.001), when all participants were analyzed together across both time points. No significant change was observed for the self‐control domain (*p* = 0.082). A significant time × group interaction was also found for total PGWB‐I scores (adjusted *p* = 0.002), indicating a greater improvement in the MBI group compared with controls. The same pattern was observed across individual domains, anxiety (*p* = 0.008), depression (*p* = 0.007), positive well‐being (*p* = 0.001), self‐control (*p* = 0.007), general health (*p* = 0.013), and vitality (*p* < 0.001), demonstrating a broad positive effect of the intervention on psychological well‐being (Figure 4).

**FIGURE 4:**
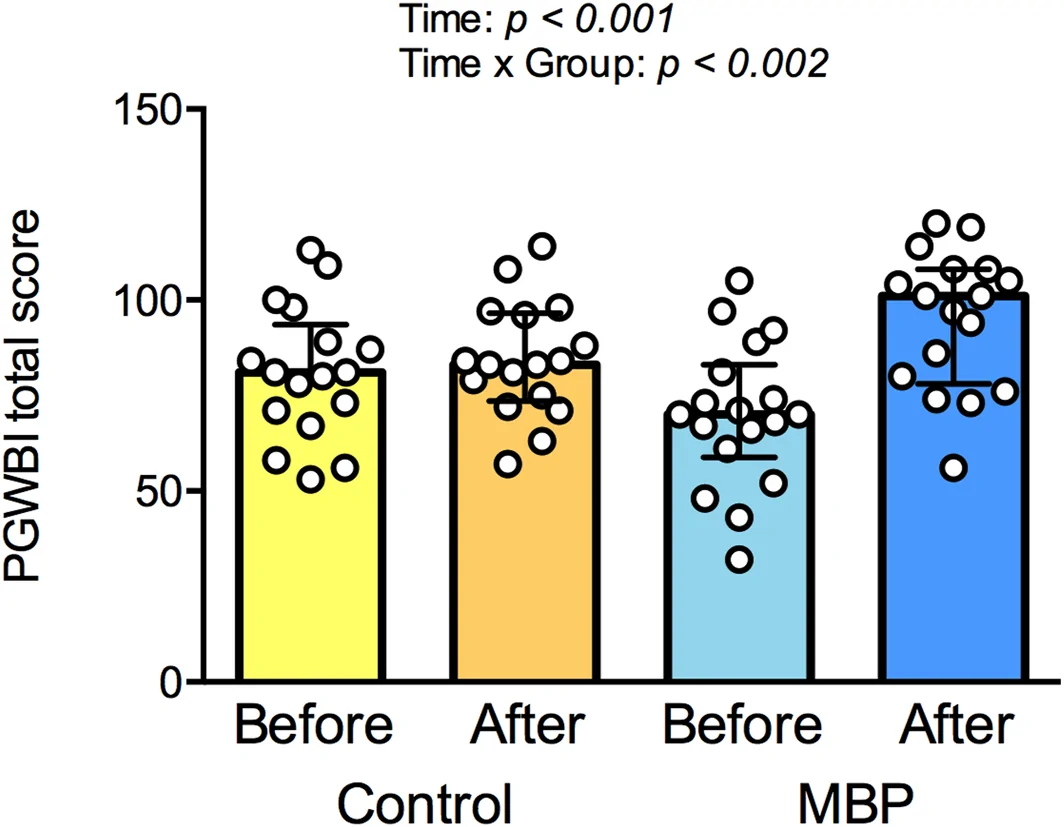
Changes in total scores of the Psychological General Well‐Being Index (PGWB‐I) before and after the mindfulness‐based intervention (MBI, *n* = 18 at baseline and *n* = 17 post‐intervention) and in the control group (*n* = 17 at baseline and post‐intervention). Bars represent medians and interquartile ranges.

In the Spearman correlation analysis, age showed a positive correlation with the change of global symptom scores (*r* = 0.593; *p* = 0.012, Figure 5A), anxiety (*r* = 0.645; *p* = 0.005, Figure 5C), and stress (*r* = 0.637; *p* = 0.006, Figure 5D). In other words, older women tended to exhibit smaller reductions in symptoms following the intervention compared with younger participants. For the depression domain, a positive correlation of moderate magnitude was observed (*r* = 0.414), but it did not reach statistical significance (*p* = 0.098), indicating only a trend toward association (Figure 5B).

**FIGURE 5:**
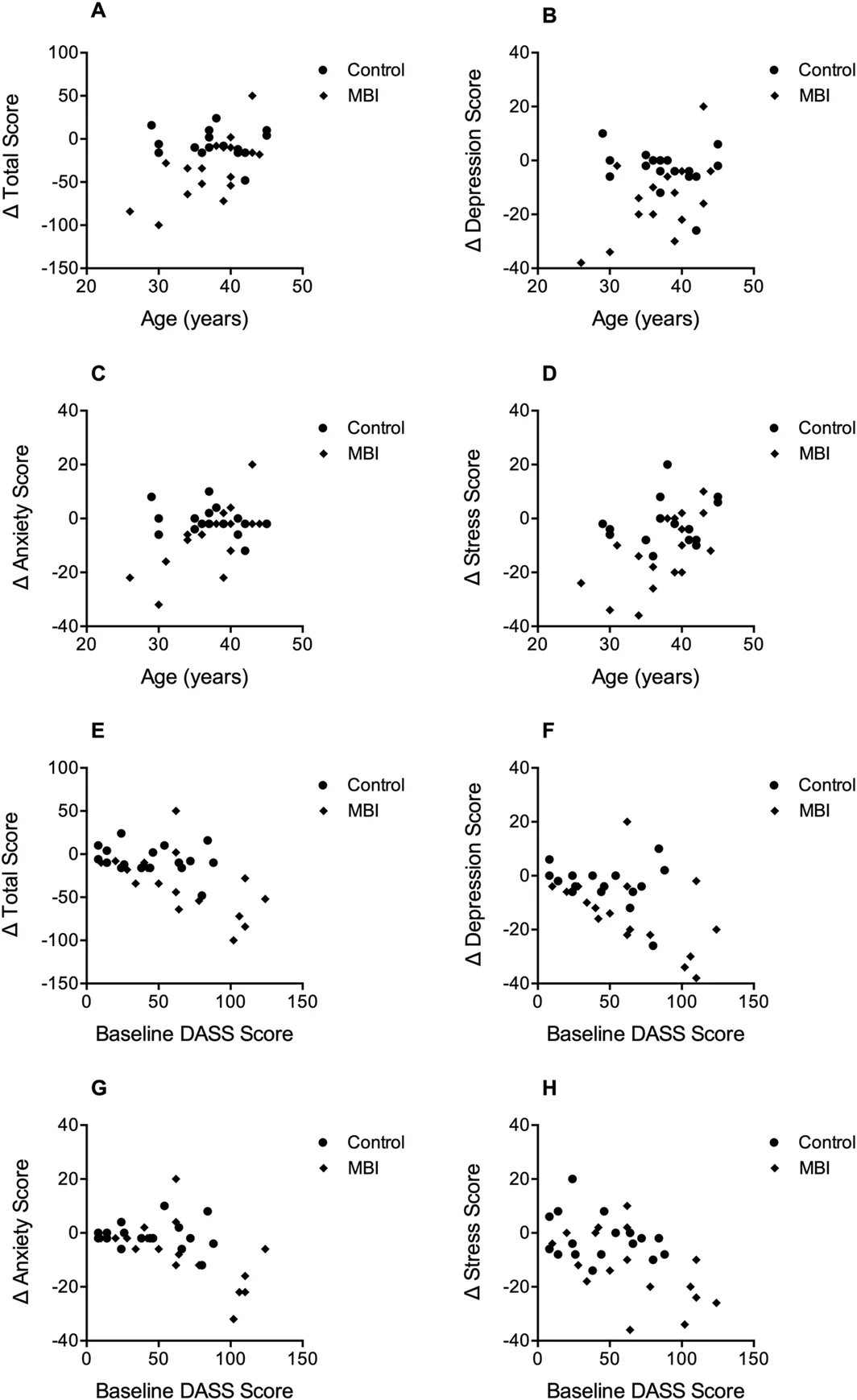
Correlation analyzes between participant's age (A–D) and basal DASS‐21 scores (E–H) and the change (⊗) in total and subscale scores between the pre‐intervention and post‐intervention assessments.

In addition, higher levels of psychological distress at baseline were negatively correlated with post‐intervention changes, meaning that higher initial scores were associated with greater reductions in total symptoms (*r* = −0.641; *p* = 0.006, Figure 5E), depression (*r* = −0.506; *p* = 0.038, Figure 5F), anxiety (*r* = −0.650; *p* = 0.005, Figure 5G), and stress (*r* = −0.551; *p* = 0.022, Figure 5H). Conversely, the number of previous miscarriages showed no significant association with the DASS‐21 outcomes for global symptoms (*r* = 0.027; *p* = 0.917), depression (*r* = −0.072; *p* = 0.784), anxiety (*r* = −0.070; *p* = 0.790), or stress (*r* = 0.137; *p* = 0.600), suggesting that the obstetric history in this regard did not influence the magnitude of the response to the intervention.

## 4 Discussion

The results of this RCT indicate that MBI may be effective in reducing self‐reported symptoms of stress and depression, as assessed by the DASS‐21 questionnaire, as well as in improving overall well‐being in women with RPL. Although the sample size was calculated based on salivary cortisol levels, defined as the primary outcome, no significant differences were observed between groups for this biomarker or for other physiological markers evaluated (salivary IgA and alpha‐amylase), nor for the anxiety domain of the DASS‐21. On the other hand, the psychological scores showed significant improvement only in the intervention group, suggesting that MBI may have a greater impact on subjective dimensions of stress than on biological indicators.

A recent systematic review examined the effects of psychological and supportive interventions on stress, anxiety, and depression in women with RPL but found no RCTs including objective biomarkers such as cortisol (San Lazaro Campillo et al. 2017). Building on this gap, the present study is the first RCT to assess the effects of MBIs using both self‐report questionnaires and physiological stress markers in this population.

In the present study, no significant changes were observed in the salivary stress biomarkers cortisol, alpha‐amylase and IgA following MBI. This absence of effect may be related to several factors. First, the intervention period of 8 weeks may not have been long enough to produce modifications in stress biomarkers. Moreover, the high interindividual variability of these biomarkers may have masked potential MBI effects. Since the sample size was based on salivary cortisol, it could lack statistical power to show small variations in alpha‐amylase or IgA levels. It is also possible that the impacts of mindfulness manifest primarily at cognitive‐emotional levels, resulting in more subtle biochemical repercussions that may only become detectable over the medium or long term.

Another important point to note concerns the interpretation of basal morning cortisol. Analyses based on single samples have limitations, as they do not capture the awakening response, diurnal decline, or acute stress reactivity, and changes in raw cortisol measures are more difficult to detect (Sanada et al. 2016). Therefore, in this study, salivary cortisol was considered only as a basal morning marker, without allowing for a complete characterization of the HPA axis dynamics. Thus, the absence of diurnal assessment may have reduced sensitivity to detect subtle biological changes. Therefore, null biomarker findings should be interpreted with caution. Future investigations using multiple collections throughout the day (including awakening response and nighttime samples) are essential to confirm and expand these findings.

We found a significant time × group interaction in several psychological variables assessed through the DASS‐21, suggesting that the intervention group showed greater improvements over time compared to the control group. Significant time × group interactions were observed for the total DASS‐21 score as well as for the depression and the stress domains, indicating a greater reduction in symptoms among participants who underwent MBI. In the depression domain, a significant reduction in scores was observed in the intervention group, a finding consistent with previous studies reporting the positive impact of mindfulness on reducing depressive symptoms in women with early pregnancy loss (Nasrollahi et al. 2022), infertility (G. Wang et al. 2023) and in other populations experiencing persistent psychological distress (Goyal et al. 2014; Hofmann et al. 2010). The practice of mindfulness, by promoting greater emotional awareness and acceptance, may be associated with mood regulation and a decrease in negative rumination, factors commonly linked to depression.

In the stress domain, a reduction in scores was also identified in the intervention group. This improvement is consistent with research demonstrating the effect of mindfulness in lowering perceived stress levels among women with early pregnancy loss (Nasrollahi et al. 2022), RPL (Jensen et al. 2021; G. Wang et al. 2023), infertility (Nery et al. 2019) and other populations (Creswell 2017; Rouleau et al. 2015). Considering the context of women with RPL, a group often exposed to emotional overload and at increased risk for psychiatric disorders, it is plausible that mindfulness practice contributed to reducing overall psychological distress.

Regarding anxiety, a decrease in scores was also found in the MBI group, which aligns with the literature describing the potential of mindfulness to modulate the stress response and reduce the autonomic hyperactivity typical of anxious states (Zeidan et al. 2010). However, this benefit did not differ from that observed in the control group, possibly because anxiety symptoms subsided over time due to positive expectancy or regression to the mean. In addition, the sample size may have lacked power to detect small differences between groups regarding this secondary outcome. Furthermore, lower baseline anxiety levels in both groups could reduce the magnitude of measurable change. Finally, the MBI may primarily address the cognitive‐emotional processing related to grief and depressive rumination, rather than anticipatory anxiety.

In our study, regarding psychological well‐being, significant interactions were observed in the domains of somatic health, anxiety, depression, subjective well‐being, self‐control, perceived health, and vitality, suggesting a possible positive effect of the intervention on multiple aspects of mental health. The broad improvement observed in the PGWB‐I, encompassing different dimensions of well‐being, supports the hypothesis that the MBI may not only contribute to the reduction of negative symptoms but also promote the development of resilience, self‐control, and a positive perception of health. Although exploratory, these findings are consistent with previous studies in other clinical populations that have reported beneficial effects of MBIs across various dimensions of mental health (Trombka et al. 2021; D. N. Wang et al. 2021; G. Wang et al. 2023), and religious and existential well‐being (Nooripour et al. 2022).

In the present study, higher baseline levels of psychological distress were associated with greater reductions in depression, anxiety, and stress symptoms after the intervention, suggesting that the MBI exerted a more pronounced impact among participants with greater initial emotional burden. This pattern is consistent with previous findings that individuals with higher baseline symptom scores tend to show greater improvements following mindfulness‐based programs (Carmody and Baer 2008; Hofmann et al. 2010; Khoury et al. 2013). This trend may reflect both a larger potential for clinical change, given the greater severity of symptoms, and stronger motivation and engagement with mindfulness practices. In addition, age was negatively correlated with the magnitude of symptom reduction, indicating that younger women tended to benefit more from the intervention. This finding aligns with the meta‐analysis by which demonstrated greater effectiveness of MBIs in younger participants, possibly due to higher cognitive plasticity, emotional flexibility, and adherence to the proposed practices (Sanada et al. 2016).

Recent evidence from a RCT evaluating neurofeedback in students demonstrated significant reductions in salivary cortisol and alpha‐amylase levels, accompanied by improvements in psychological stress indicators after an 8‐week intervention (Shahyad et al. 2024). Although neurofeedback and MBI differ in their therapeutic mechanisms, these findings suggest that interventions targeting self‐regulation and stress awareness may exert beneficial effects on psychophysiological pathways, including the HPA axis and autonomic nervous system.

Our study presents some limitations. Since there was no follow‐up after the end of the intervention, the long‐term maintenance of MBI effects could not be assessed. Another limitation concerns the potential for response bias, as psychological symptoms were measured using self‐reported questionnaires. In addition, adherence to informal mindfulness practices, which could influence the magnitude of observed effects, was not evaluated. There was also no systematic quantification of adherence to home‐based mindfulness practice, which may influence intervention outcomes. Because the study was conducted in a single center, with its own socioeconomic and cultural characteristics, the generalizability of our findings is limited. It should be acknowledged that expectancy bias may have influenced self‐reported psychological outcomes, as blinding was not feasible in this behavioral intervention. Although validated instruments were used and both groups received comparable clinical follow‐up, participants in the MBI group may have anticipated emotional benefits. Therefore, non‐specific effects, including expectancy, therapist attention, group interaction, and social support, cannot be fully excluded, and future RCTs with active control groups are needed to better distinguish intervention‐specific effects from non‐specific influences. Additionally, because the sample size calculation was based on the primary biomarker outcome (salivary cortisol), the study may have been underpowered for secondary psychometric outcomes, especially domain‐specific analyses with greater variability. Furthermore, the evaluation of multiple secondary and exploratory outcomes (correlation analyses) may have increased the risk of type I error. Therefore, the positive findings observed in self‐reported psychological measures should be interpreted cautiously as exploratory and hypothesis‐generating rather than as definitive evidence of intervention efficacy.

Finally, the biomarkers analyzed are sensitive to various external factors, which could affect the reproducibility of the results. Regarding salivary cortisol assessment, it was performed using a single morning sample (8:00–9:00 a.m.) under rigorously standardized conditions for fasting, prior rest, and use of collection tubes. Although this approach minimized potential sources of variability, it represented a raw measure of basal secretion and did not capture cortisol awakening response, diurnal slope, or total daily cortisol output, particularly because individual awakening times were not recorded. Therefore, this single‐point assessment may have had limited sensitivity for detecting subtle HPA‐axis changes and did not fully reflect chronic stress load (Sanada et al. 2016). Accordingly, the absence of significant biomarker changes should be interpreted cautiously.

In summary, this study did not demonstrate significant effects of MBI on the prespecified primary biomarker outcomes, including salivary cortisol, IgA, and alpha‐amylase. However, improvements in secondary self‐reported psychological outcomes suggest potential psychosocial benefits of MBIs for women with RPL. These findings are exploratory and require confirmation in larger, adequately powered studies with active control groups and longer follow‐up. If confirmed, such interventions may represent a valuable complementary approach to addressing the emotional and psychosocial burden associated with RPL.

## Funding

Research supported by Conselho Nacional de Desenvolvimento Científico e Tecnológico (CNPq) through the National Institute of Hormones and Women's Health (grant # 465482/2014‐7).

## Conflicts of Interest

The authors declare no conflicts of interest.

## Data Availability

The data that support the findings of this study are available from the corresponding author upon reasonable request.

## References

[smi70228-bib-0001] Aguilar‐Raab, C. , M. Stoffel , C. Hernández , et al. 2021. “Effects of a Mindfulness‐Based Intervention on Mindfulness, Stress, Salivary Alpha‐Amylase and Cortisol in Everyday Life.” Psychophysiology 58, no. 12: e13937. 10.1111/psyp.13937. 34525214

[smi70228-bib-0002] Bergner, A. , R. Beyer , B. F. Klapp , and M. Rauchfuss . 2008. “Pregnancy After Early Pregnancy Loss: A Prospective Study of Anxiety, Depressive Symptomatology and Coping.” Journal of Psychosomatic Obstetrics and Gynaecology 29, no. 2: 105–113. 10.1080/01674820701687521. 17943588

[smi70228-bib-0003] Blair, J. , J. Adaway , B. Keevil , and R. Ross . 2017. “Salivary Cortisol and Cortisone in the Clinical Setting.” Current Opinion in Endocrinology Diabetes and Obesity 24, no. 3: 161–168. 10.1097/MED.0000000000000328. 28375882

[smi70228-bib-0004] Carmody, J. , and R. A. Baer . 2008. “Relationships Between Mindfulness Practice and Levels of Mindfulness, Medical and Psychological Symptoms and Well‐Being in a Mindfulness‐Based Stress Reduction Program.” Journal of Behavioral Medicine 31, no. 1: 23–33. 10.1007/s10865-007-9130-7. 17899351

[smi70228-bib-0005] Carp, H. J. , ed. 2014. Recurrent Pregnancy Loss: Causes, Controversies, and Treatment. CRC Press.

[smi70228-bib-0006] Chojnowska, S. , I. Ptaszyńska‐Sarosiek , A. Kępka , M. Knaś , and N. Waszkiewicz . 2021. “Salivary Biomarkers of Stress, Anxiety and Depression.” Journal of Clinical Medicine 10, no. 3: 517. 10.3390/jcm10030517. PMC7867141 33535653

[smi70228-bib-0007] Creswell, J. D. 2017. “Mindfulness Interventions.” Annual Review of Psychology 68, no. 1: 491–516. 10.1146/annurev-psych-042716-051139. 27687118

[smi70228-bib-0008] Dunlop, W. P. , J. M. Cortina , J. B. Vaslow , and M. J. Burke . 1996. “Meta‐Analysis of Experiments With Matched Groups or Repeated Measures Designs.” Psychological Methods 1: 170–177. 10.1037/1082-989X.1.2.170.

[smi70228-bib-0009] Dupuy, H. J. , N. K. Wenger , M. E. Mattson , C. D. Furberg , and J. Elinson . 1984. Assessment of Quality of Life in Clinical Trials of Cardiovascular Therapies. Le Jacq. 10.1016/s0002-9149(84)80232-5 6333175

[smi70228-bib-0010] Engeland, C. G. , J. A. Bosch , and N. Rohleder . 2019. “Salivary Biomarkers in Psychoneuroimmunology.” Current Opinion in Behavioral Sciences 28: 58–65. 10.1016/j.cobeha.2019.01.007. PMC7094032 32215283

[smi70228-bib-0011] Espírito Santo, R. P. S. 2015. Bem‐estar Psicológico: Validação da escala Psychological General Well‐Being Para a População Portuguesa [Dissertação de Mestrado, Universidade Lusófona do Porto].

[smi70228-bib-0012] Gasnier, M. , A. Pelissolo , G. Bondolfi , et al. 2017. “Les interventions basées sur la pleine conscience dans le trouble obsessionnel compulsif: mécanismes d’action et présentation d’une étude pilote [Mindfulness‐Based Interventions in Obsessive‐Compulsive Disorder: Mechanisms of action and Presentation of a Pilot Study].” L'Encephale 43, no. 6: 594–599. 10.1016/j.encep.2016.10.004. 27887679

[smi70228-bib-0013] Goyal, M. , S. Singh , E. M. Sibinga , et al. 2014. “Meditation Programs for Psychological Stress and Well‐Being: A Systematic Review and Meta‐Analysis.” JAMA Internal Medicine 174, no. 3: 357–368. 10.1001/jamainternmed.2013.13018. PMC4142584 24395196

[smi70228-bib-0014] Gröschl, M. 2008. “Current Status of Salivary Hormone Analysis.” Clinical Chemistry 54, no. 11: 1759–1769. 10.1373/clinchem.2008.108910. 18757583

[smi70228-bib-0015] Grossi, E. , N. Groth , P. Mosconi , et al. 2006. “Development and Validation of the Short Version of the Psychological General Well‐Being Index (PGWB‐S).” Health and Quality of Life Outcomes 4, no. 1: 88. 10.1186/1477-7525-4-88. PMC1647268 17105655

[smi70228-bib-0016] Hassanvandi, S. , R. Mohammadzadegan , S. H. Saadat , B. Hatef , and S. Shahyad . 2025. “Effectiveness of Mindfulness‐Based Stress Reduction on Salivary Cortisol and α‐Amylase Level, Anxiety and Depression in Students: A Randomized and Parallel‐Group Clinical Trial.” Applied Psychophysiology and Biofeedback 50, no. 3: 417–428. 10.1007/s10484-025-09690-5. 40694304

[smi70228-bib-0017] He, L. , T. Wang , H. Xu , et al. 2019. “Prevalence of Depression and Anxiety in Women With Recurrent Pregnancy Loss and the Associated Risk Factors.” Archives of Gynecology and Obstetrics 300, no. 4: 1061–1066. 10.1007/s00404-019-05264-z. 31485778

[smi70228-bib-0018] Hedegaard, S. , S. K. Landersoe , L. R. Olsen , M. C. Krog , A. M. Kolte , and H. S. Nielsen . 2021. “Stress and Depression Among Women and Men Who Have Experienced Recurrent Pregnancy Loss: Focusing on Both Sexes.” Reproductive BioMedicine Online 42, no. 6: 1172–1180. 10.1016/j.rbmo.2021.03.012. 33962908

[smi70228-bib-0019] Hoffman, C. J. , S. J. Ersser , J. B. Hopkinson , P. G. Nicholls , J. E. Harrington , and P. W. Thomas . 2012. “Effectiveness of Mindfulness‐based Stress Reduction in Mood, Breast‐ and Endocrine‐Related Quality of Life, and well‐being in Stage 0 to III Breast Cancer: A Randomized, Controlled Trial.” Journal of Clinical Oncology: Official journal of the American Society of Clinical Oncology 30, no. 12: 1335–1342. 10.1200/JCO.2010.34.0331. 22430268

[smi70228-bib-0020] Hofmann, S. G. , A. T. Sawyer , A. A. Witt , and D. Oh . 2010. “The Effect of Mindfulness‐Based Therapy on Anxiety and Depression: A Meta‐Analytic Review.” Journal of Consulting and Clinical Psychology 78, no. 2: 169–183. 10.1037/a0018555. PMC2848393 20350028

[smi70228-bib-0021] Inder, W. J. , G. Dimeski , and A. Russell . 2012. “Measurement of Salivary Cortisol in 2012—Laboratory Techniques and Clinical Indications.” Clinical Endocrinology 77, no. 5: 645–651. 10.1111/j.1365-2265.2012.04508.x. 22812714

[smi70228-bib-0022] Jensen, K. H. K. , M. C. Krog , E. Koert , et al. 2021. “Meditation and Mindfulness Reduce Perceived Stress in Women With Recurrent Pregnancy Loss: A Randomized Controlled Trial.” Reproductive BioMedicine Online 43, no. 2: 246–256. 10.1016/j.rbmo.2021.04.018. 34112605

[smi70228-bib-0023] Kabat‐Zinn, J. 2003. “Mindfulness‐Based Interventions in Context: Past, Present, and Future.” Clinical Psychology: Science and Practice 10, no. 2: 144–156. 10.1093/clipsy.bpg016.

[smi70228-bib-0024] Khoury, B. , T. Lecomte , G. Fortin , et al. 2013. “Mindfulness‐Based Therapy: A Comprehensive Meta‐Analysis.” Clinical Psychology Review 33, no. 6: 763–771. 10.1016/j.cpr.2013.05.005. 23796855

[smi70228-bib-0025] Kirschbaum, C. , and D. H. Hellhammer . 1989. “Salivary Cortisol in Psychobiological Research: An Overview.” Neuropsychobiology 22, no. 3: 150–169. 10.1159/000118611. 2485862

[smi70228-bib-0026] Klock, S. C. , G. Chang , A. Hiley , and J. Hill . 1997. “Psychological Distress Among Women With Recurrent Spontaneous Abortion.” Psychosomatics 38, no. 5: 503–507. 10.1016/S0033-3182(97)71428-2. 9314720

[smi70228-bib-0027] Kolte, A. M. , L. R. Olsen , E. M. Mikkelsen , O. B. Christiansen , and H. S. Nielsen . 2015. “Depression and Emotional Stress Is Highly Prevalent Among Women With Recurrent Pregnancy Loss.” Human Reproduction 30, no. 4: 777–782. 10.1093/humrep/dev014. PMC4359400 25662810

[smi70228-bib-0028] Lovibond, S. H. , and P. F. Lovibond . 1995. Manual for the Depression Anxiety & Stress Scales. 2nd ed. Psychology Foundation.

[smi70228-bib-0029] Lynch, S. , M. L. Gander , N. Kohls , B. Kudielka , and H. Walach . 2011. “Mindfulness‐Based Coping With University Life: A Non‐randomized Wait‐List‐Controlled Pilot Evaluation.” Stress and Health 27, no. 5: 365–375. 10.1002/smi.1382.

[smi70228-bib-0030] Nasrollahi, M. , M. Ghazanfar Pour , A. Ahmadi , M. Mirzaee , and K. Alidousti . 2022. “Effectiveness of Mindfulness‐Based Stress Reduction on Depression, Anxiety, and Stress of Women With the Early Loss of Pregnancy in Southeast Iran: A Randomized Control Trial.” Reproductive Health 19, no. 1: 233. 10.1186/s12978-022-01543-2. PMC9801648 36581926

[smi70228-bib-0031] Nater, U. M. , and N. Rohleder . 2009. “Salivary Alpha‐Amylase as a Non‐Invasive Biomarker for the Sympathetic Nervous System: Current State of Research.” Psychoneuroendocrinology 34, no. 4: 486–496. 10.1016/j.psyneuen.2009.01.014. 19249160

[smi70228-bib-0032] Nery, S. F. , S. P. C. Paiva , É. L. Vieira , et al. 2019. “Mindfulness‐Based Program for Stress Reduction in Infertile Women: Randomized Controlled Trial.” Stress and Health: journal of the International Society for the Investigation of Stress 35, no. 1: 49–58. 10.1002/smi.2839. 30328241

[smi70228-bib-0033] Nooripour, R. , S. Hosseinian , M. Sobhaninia , et al. 2022. “Predicting Fear of COVID‐19 Based on Spiritual Well‐Being and Self‐Efficacy in Iranian University Students by Emphasizing the Mediating Role of Mindfulness.” Journal of Practice in Clinical Psychology 10, no. 1: 1–10. 10.32598/jpcp.10.1.288.6.

[smi70228-bib-0034] O'Leary, K. , S. O'Neill , and S. Dockray . 2016. “A Systematic Review of the Effects of Mindfulness Interventions on Cortisol.” Journal of Health Psychology 21, no. 9: 2108–2121. 10.1177/1359105315569095. 25673371

[smi70228-bib-0035] Paiva, S. P. C. , S. F. Nery , E. B. Magalhães , et al. 2016. “Impact of a Short‐Term, Mindfulness‐Based Stress Reduction Program on the Well‐Being of Infertile Women: A Mixed‐Method Study.” Journal of Endometriosis and Pelvic Pain Disorders 7, no. 4: 136–140. 10.5301/je.5000227.

[smi70228-bib-0036] Rai, R. , and L. Regan . 2006. “Recurrent Miscarriage.” Lancet (London, England) 368, no. 9535: 601–611. 10.1016/S0140-6736(06)69204-0. 16905025

[smi70228-bib-0037] Rouleau, C. R. , S. N. Garland , and L. E. Carlson . 2015. “The Impact of Mindfulness‐Based Interventions on Symptom Burden, Positive Psychological Outcomes, and Biomarkers in Cancer Patients.” Cancer Management and Research 7: 121–131. 10.2147/CMAR.S64165. PMC4457221 26064068

[smi70228-bib-0038] Sanada, K. , J. Montero‐Marin , M. Alda Díez , et al. 2016. “Effects of Mindfulness‐Based Interventions on Salivary Cortisol in Healthy Adults: A Meta‐Analytical Review.” Frontiers in Physiology 7: 471. 10.3389/fphys.2016.00471. PMC5069287 27807420

[smi70228-bib-0039] San Lazaro Campillo, I. , S. Meaney , K. McNamara , and K. O'Donoghue . 2017. “Psychological and Support Interventions to Reduce Levels of Stress, Anxiety or Depression on Women's Subsequent Pregnancy With a History of Miscarriage: An Empty Systematic Review.” BMJ Open 7, no. 9: e017802. 10.1136/bmjopen-2017-017802. PMC5595175 28882928

[smi70228-bib-0040] Saraiva, E. M. , J. M. S. Fortunato , and C. Gavina . 2005. “Oscilações do Cortisol na depressão e sono/vigília.” Revista Portuguesa de Psicossomática 7, no. 1–2: 89–100.

[smi70228-bib-0041] Saunders, P. A. , R. E. Tractenberg , R. Chaterji , et al. 2007. “Promoting self‐Awareness and Reflection Through an Experiential Mind‐Body Skills Course for First Year Medical Students.” Medical Teacher 29, no. 8: 778–784. 10.1080/01421590701509647. PMC4372185 17852720

[smi70228-bib-0042] Segerstrom, S. C. , and G. E. Miller . 2004. “Psychological Stress and the Human Immune System: A Meta‐Analytic Study of 30 Years of Inquiry.” Psychological Bulletin 130, no. 4: 601–630. 10.1037/0033-2909.130.4.601. PMC1361287 15250815

[smi70228-bib-0043] Shahyad, S. , E. Rahmani , M. Nikdanesh , A. Ashoori , S. Azadi , and S. Hassanvandi . 2024. “Effectiveness of Neurofeedback on Psychological Stress, Salivary Cortisol and α‐Amylase Level in Students: A Randomized and Parallel‐Group Clinical Trial.” Iranian Journal of Psychiatry and Behavioral Sciences 18, no. 1: e140330. 10.5812/ijpbs-140330.

[smi70228-bib-0044] Trombka, M. , M. Demarzo , D. Campos , et al. 2021. “Mindfulness Training Improves Quality of Life and Reduces Depression and Anxiety Symptoms Among Police Officers: Results From the POLICE Study‐A Multicenter Randomized Controlled Trial.” Frontiers in Psychiatry 12: 624876. 10.3389/fpsyt.2021.624876. PMC7952984 33716824

[smi70228-bib-0045] Tulandi, T. , and H. M. Al‐Fozan . 2024. “Recurrent Pregnancy Loss: Definition and Etiology.” UpToDate: Retrieved August 9, 2024, from. https://www.uptodate.com/contents/recurrent‐pregnancy‐loss‐definition‐and‐etiology.

[smi70228-bib-0046] Vargas‐Uricoechea, H. , A. Castellanos‐Pinedo , K. Urrego‐Noguera , et al. 2024. “Mindfulness‐Based Interventions and the Hypothalamic‐Pituitary‐Adrenal Axis: A Systematic Review.” Neurology International 16, no. 6: 1552–1584. 10.3390/neurolint16060115. PMC11587421 39585074

[smi70228-bib-0047] Wang, D. N. , X. L. Weng , and L. L. Gao . 2021. “Mindfulness‐Based Intervention in Chinese Pregnant Women With Recurrent Miscarriage: A Non‐Randomized Controlled Study.” Midwifery 103: 103152. 10.1016/j.midw.2021.103152. 34601233

[smi70228-bib-0048] Wang, G. , X. Liu , and J. Lei . 2023. “Effects of Mindfulness‐Based Intervention for Women With Infertility: A Systematic Review and Meta‐Analysis.” Archives of Women's Mental Health 26, no. 2: 245–258. 10.1007/s00737-023-01307-2. 36952004

[smi70228-bib-0049] Wenger, N. K. , M. E. Mattson , C. D. Furberg , and J. Elinson . 1984. “Assessment of Quality of Life in Clinical Trials of Cardiovascular Therapies.” American Journal of Cardiology 54, no. 7: 908–913. 10.1016/s0002-9149(84)80232-5. 6333175

[smi70228-bib-0050] Zeidan, F. , S. K. Johnson , B. J. Diamond , Z. David , and P. Goolkasian . 2010. “Mindfulness Meditation Improves Cognition: Evidence of Brief Mental Training.” Consciousness and Cognition 19, no. 2: 597–605. 10.1016/j.concog.2010.03.014. 20363650

